# Outcomes after thumb carpometacarpal joint stabilization with an abductor pollicis longus tendon strip for the treatment of chronic instability

**DOI:** 10.1007/s00402-019-03302-8

**Published:** 2019-11-06

**Authors:** Alexandra Stauffer, Yvonne Schwarz, Marion Uranyi, Florian Schachinger, Werner Girsch, Rudolf Ganger, Sebastian Farr

**Affiliations:** 1 Department of Pediatric Orthopaedics and Adult Foot and Ankle Surgery, Orthopedic Hospital Vienna-Speising; Affiliated to Medical University Vienna, Speisingerstrasse 109, 1130 Vienna, Austria; 2 Department of Physical Therapy and Rehabilitation, Orthopedic Hospital Speising, Vienna, Austria; 3 Department for Plastic Surgery, Medical University Graz, Graz, Austria

**Keywords:** Carpometacarpal joint, CMC joint, Chronic instability, Habitual dislocation, Stabilization, Abductor pollicis longus

## Abstract

**Introduction:**

Instabilities of the thumb carpometacarpal (CMC) joint, caused by idiopathic ligamentous hyperlaxity, trauma or other conditions may lead to pain, functional impairment and eventually osteoarthritis. Several techniques have been described to enhance stability of the CMC 1. The aim of this study was to evaluate postoperative outcomes after CMC 1 joint stabilization using a soft-tissue procedure in patients with chronic instability.

**Materials and methods:**

This study was designed as a retrospective study with a single follow-up visit after a minimum of 1 year postoperatively. All patients who underwent stabilization of the CMC 1 with an abductor pollicis longus (APL) tendon strip for chronic, habitual instability were re-assessed using clinical examination, dedicated outcome scores [Visual Analogue Scale (VAS); The Disability of the Arm, Shoulder and Hand (DASH) score; Nelson score; Kapandji opposition score], grip and pinch strength measurements, and radiographic examination.

**Results:**

12 patients (15 operated thumbs) with a mean age at surgery of 23.2 (± 9.3) years were included after a mean follow-up period of 3.5 (± 1.3) years. The postoperative outcomes indicated excellent results, with a mean DASH score of 13.3 (± 11.3), VAS 1.1 at rest (and 2.8 during stress) and Nelson score of 87.7 (± 11.3). Postoperative grip, pinch strength and passive stability were not significantly different between operated and non-operated sides (*p* = 0.852; *p* = 0.923 and *p* = 0.428, respectively). We observed one case of recurrent instability besides no other complications. However, patients with trapezium hypoplasia (5 of 12) were more prone to signs of radiographic instability during stress testing.

**Conclusions:**

Thumb carpometacarpal stabilization with an APL tendon strip yielded excellent clinical outcomes and low morbidity in the mid-term. However, long-term follow-up is needed to assess specifically whether patients with trapezium hypoplasia may be more prone to clinical symptom recurrence than those with normal anatomy.

**Level of evidence:**

Level IV

## Introduction

The carpometacarpal (CMC) joint is the most important functional joint of the first digit. It is a saddle joint, thereby allowing for a wide range of motion, however, at the cost of joint stability. According to biomechanical studies, forces and loads on this joint are higher compared to more distally located finger joints [1]. Instabilities of the CMC 1 joint, caused by idiopathic laxity of the ligaments, trauma, overexertion or other conditions such as dysplasia of the trapezium, may lead to excessive and aberrant mobility due to gross abnormal alignment. To our experience, CMC 1 instability may occur in patients with normal trapezium anatomy due to ligamentous laxity as the primary pathogenic factor. However, dysplasia of the trapezium may often (but not always) be an underlying, aggravating factor for the development of instability. In such cases, an increased radial tilt of the articular surface (> 40° in relation to the metacarpal 2) with a concomitant decrease of trapezium width may be present. As a consequence, such misalignments may result in fixation and subsequent deformity of unstable joints over time [2].

Clinical findings of instability of the CMC 1 joint include pain, functional limitations, subluxation and loss of strength, most commonly exacerbated by the performance of a pinch grip. Laxities of ligaments located at the basal joint of the thumb cause dorso-radial translation at the base of the metacarpal 1, which seems to be one of the main effectors of osteoarthritis (OA) [3]. There seems to be an association between joint hypermobility caused by laxities of ligaments and the presence and severity of CMC 1 OA [4]. OA of the hand is a common hereditary condition that primarily occurs in postmenopausal women. This condition is associated with substantial morbidity even though it is not considered a serious disorder [5]. Therefore, early reconstruction of ligaments in unstable joints may presumably reduce the risk of joint degeneration and OA.

In 1973, Eaton and Littler originally proposed the technique of ligament reconstruction involving the weaving of a slip of the flexor carpi radialis (FCR) through the first metacarpal and around the abductor pollicis longus (APL) and FCR tendon [6]. One of the more recently reported surgical approaches is the reduction of the CMC 1 joint, followed by transosseous ligament reconstruction using a distal pedicled tendon strip from the APL muscle to achieve a satisfactory stability of the joint [3].

However, in the presence of osteoarthritis of the basal thumb joint, other surgical approaches compared to ligament reconstruction techniques seem to improve symptoms especially in young manual workers. Pillukat et al. evaluated the arthrodesis of the trapeziometacarpal joint in regard to strength, stability and pain reduction [7]. They resected the articular surfaces of the trapeziometacarpal joint using a dorsal approach to apply a dorsal T-shaped plate. Postoperative results showed a high degree of patient satisfaction, as well as improvement of clinical symptoms.

The aim of this study was to evaluate postoperative clinical and radiographic outcomes after CMC 1 joint stabilization in patients with chronic instability using a soft-tissue stabilization procedure. We hypothesized that our proposed technique will lead to restoration of CMC 1 stability.

## Patients and methods

### Patient selection

**Fig. 1:**
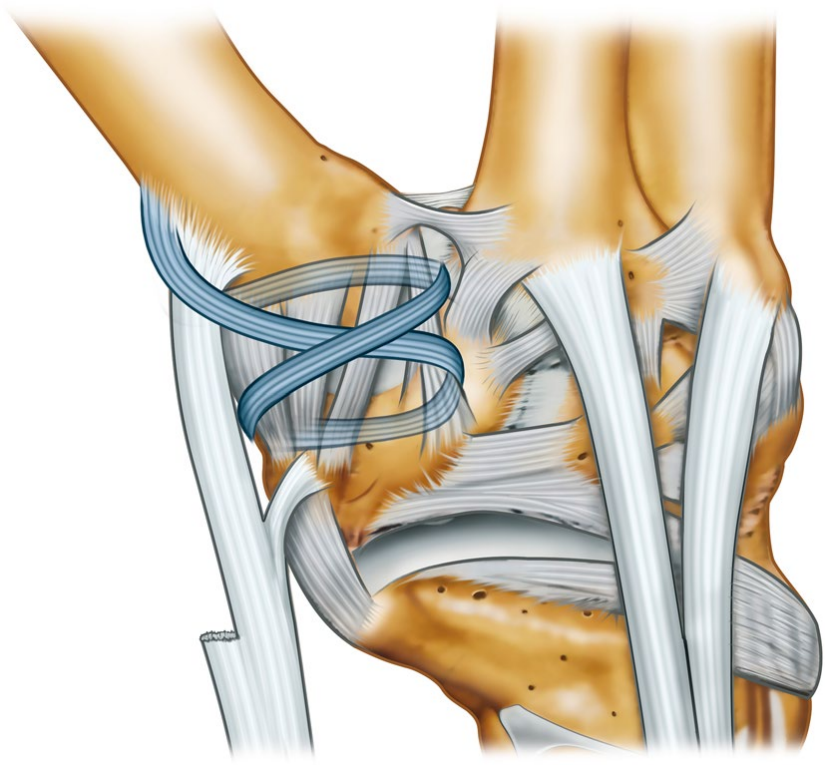
Schematic drawing of the APL tendon strip routing used for soft-tissue stabilization of the CMC 1

This study was designed as a retrospective study (Level IV) with a single follow-up visit of the study cohort. Institutional Review Board (IRB) approval was obtained prior to data acquisition. The hospital’s database was manually screened for patients undergoing stabilization of the CMC 1 with an APL tendon strip procedure for chronic instability between 2009 and 2018 to provide a minimum follow-up of 1 year. Surgery is usually indicated in cases with therapy-refractory thumb pain due to habitual CMC 1 instability. Inclusion criteria were thus patients with chronic instability of the CMC 1 in adolescence or adulthood, who underwent a soft-tissue stabilization procedure (Fig. 1) without additional concomitant surgical interventions. Patients with additional surgical procedures (i.e., arthroscopy) and children under the age of 6 years were excluded. After extraction of appropriate cases, patients were contacted via telephone and asked for a single re-evaluation of their current hand status.

### Patient evaluation

All included patients were both clinically and radiographically evaluated during a single visit in our hospital. Informed consent was obtained from all individual participants included in the study. An independent investigator, who was not involved in the surgery, assessed demographic data (i.e., age, weight, BMI), postoperative complications and clinical parameters (passive CMC 1 shift test; grade 0–3). Clinical data were organized using validated functional outcome scores [(1) Visual Analogue Scale (VAS, 0–10) for pain at rest and during stress; (2) the Disability of the Arm, Shoulder and Hand (DASH) score (0–100); (3) the Nelson score for basal OA of the thumb (0–100)]; thumb range of motion including the Kapandji opposition score (1–10), grip and pinch strength measurements using the Jamar Dynamometer (Biometrics Ltd., UK), which were completed by another independent investigator. The DASH score is a common questionnaire consisting of 30 questions, which measures the general outcome of upper extremity pathologies [8]. The Nelson score, however, is a recently developed 10-question score, which specifically assesses the outcome of thumb pain. This score is reversed with 0 being considered asymptomatic and 100 being the most severe pain thus correlating with the DASH score [9].

Radiographic evaluation included (1) dorsopalmar (DP) (2) zither-player projection [10] and (3) bilateral DP stress test radiographs of the thumb ray. For the latter, the thumbs were positioned parallel to each other in forearm pronation, with the patient pushing the thumb tips radially strongly against each other. All radiographs were interpreted and rated according to instability severity. The bilateral DP stress test was specifically used to examine the postoperative CMC 1 shift compared to the contralateral side. We therefore classified the radiographic stress test findings into four groups: no shift (up to 20% of physiological shift of the metacarpal I base in relation to the trapezium), mild (20–40%), moderate (40–60%) and severe CMC 1 dislocation (more than 60%). We moreover assessed whether the trapezium was hypoplastic or not [11]. These cases are characterized by an increased slope/radial tilt of the distal articular trapezium surface and/or reduced width of the bone.

### Surgical technique

**Fig. 2:**
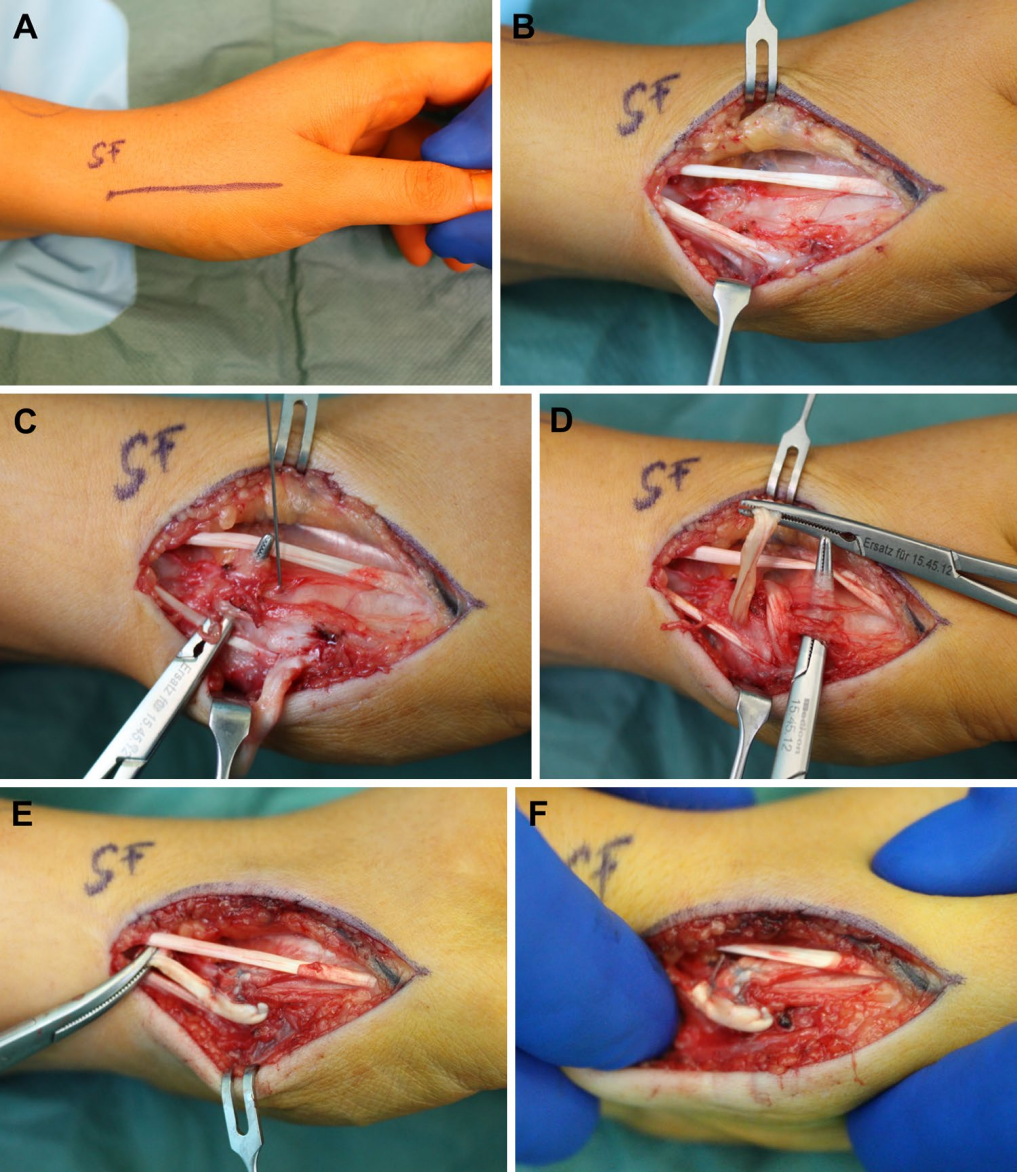
Intraoperative situs is shown. **a** A straight incision is drawn centered over the CMC 1, **b** the APL tendon is followed to its insertion, **c** transverse tunnels are made with a small clamp, **d** the tendon strip is pulled through the tunnels (figure-of-eight), **e** the knots are secured, and **f** stability is confirmed bimanually

A 5–6 cm longitudinal incision, centered dorso-radially over the CMC 1 joint is marked (Fig. 2a). Careful dissection is performed, and, after division of the first extensor tendon sheath, the APL tendon is mobilized and followed distally down to its insertion (Fig. 2b). The CMC 1 joint and capsule are clinically verified. After sharp incision, two deep transverse tunnels (1 proximal, 1 distal to the CMC joint) are created at the dorsal aspect using a blunt mosquito clamp (Fig. 2c). The tunnels should lie deep to the joint capsule and ligaments and should be at least 12–15 mm apart from each other. Thereafter, half of the APL tendon is harvested proximally and this distally based tendon strip is then reflected. The strip is pulled through the tunnels in a figure-of-eight fashion (Fig. 2d) and several FibreWire (Arthrex Inc., Naples) sutures are placed to secure the knot (Fig. 2e). A passive shift test is eventually performed to verify restored stability (Fig. 2f). A short-arm thumb cast is applied for 6 weeks followed by a thermoplastic thumb splint and occupational therapies. Sports activities with manual involvement were not allowed until 12 weeks postoperatively.

### Statistical analysis

All continuous data are reported using means, standard deviation (SD) and ranges. Postoperative grip and pinch measurements of the operated thumb and the contralateral, non-operated side were compared using independent *t* tests after data normality was confirmed by the Kolmogorov–Smirnov test. Furthermore, non-parametric data of both groups (postoperative clinical and radiologic shift tests) were compared using the Mann–Whitney *U* test. A *p* value of < 0.05 was considered statistically significant. All calculations were performed with SPSS version 23.0 (SPSS Inc., Chicago, IL, USA).

## Results

### Demographic data

We initially considered a total number of 24 patients, who met the relevant inclusion criteria after chart review, to be appropriate for inclusion. 12 patients (15 operated thumbs) could eventually be reached for clinical and radiological follow-up (Table 1). The mean age at surgery was 23.2 years (±9.3 years, range 7.4–41.3 years); the mean age at re-evaluation of our patients was 26.7 years (±9.4 years, range 11.0–43.5 years). The mean follow-up period between operation and re-evaluation was 3.5 years (± 1.3 years, range 1.3–5.8 years). Three patients underwent surgery on their left thumb, six on the right, and three patients had surgery on both hands, respectively (Table 1).

**Table 1:** Demographic details of the study group

Variable	Case group
Total procedures, *n*	15
Patient characteristics	
Sex, male, *n*	1
Sex, female, *n*	11
Affected thumb, right, *n*	9
Affected thumb, left, *n*	6
BMI, kg/m^2^	24.3 (17.3–44.1)
Follow-up characteristics	
Age at surgery, years	23.2 (7.4–41.3)
Age at re-evaluation, years	26.7 (11.0–43.5)
Follow-up period, years	3.5 (1.3–5.8)

Data are expressed as mean (range) unless indicated otherwise

### Clinical outcomes

**Fig. 3:**
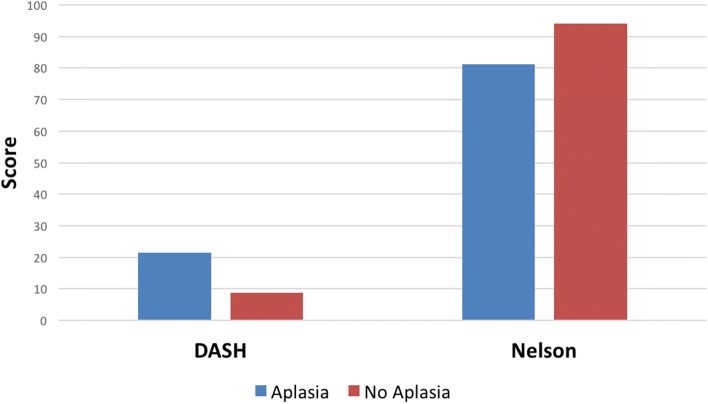
Mean values of DASH (20.1 ± 10.9 with aplasia vs. 8.8 ± 9.6 without aplasia; *p* = 0.054) and Nelson scores (78.0 ± 8.8 with aplasia vs. 94.2 ± 7.4 without aplasia; *p* = 0.002) are shown

All patients were satisfied with the outcome and would undergo the same surgery again. The mean DASH score was 13.3 (± 11.3), 13.8 (± 18.1) for the optional sports/performing arts module and 17.2 (± 28.9) for the optional work module, respectively. Postoperative VAS scores showed satisfactory results as well, with a mean of 1.1 (± 2.2) at rest and 2.8 (± 3.3) during stress. The postoperative Nelson Score resulted in a mean score of 87.7 (± 11.3) in operated hands versus 98.5 (± 4.2) on the contralateral, non-operated side. The evaluation of range of motion using the Kapanji opposition score showed a mean of 9.8 (± 0.35). Patients with trapezium hypoplasia showed slightly inferior, but still good outcomes in the DASH and Nelson scores, respectively (Fig. 3).

Grip strength measurements revealed a mean of 24.6 kg (± 5.5) in operated compared to 24.3 kg (± 4.9) in non-operated hands (*p* = 0.852), whereas pinch strength measurements showed 5.6 kg (± 1.3) for hands that underwent surgery as compared to the non-operated sides (5.4 kg ± 1.2; *p* = 0.923). The results of the postoperative CMC 1 shift test are shown in Table 2. There was no significant difference found between operated and non-operated sides (*p* = 0.428). One single patient (6.7%) reported a recurrence of instability on one operated hand 3.5 years after surgery. However, the patient elected to not undergo revision surgery because the symptoms were rated as acceptable. There were no other surgical complications such as numbness, bleeding or wound healing problems reported after the procedure.

**Table 2:** Outcomes of postoperative clinical and radiographic stress testing of stability

Case	Side	Operated vs. non-operated	Clinical shift	Radiographic shift	Trapezium hypoplasia
1	R	Non-OP	3	2	None
	L	OP	2	2	None
2	R	OP	0	1	None
	L	OP	0	1	None
3	R	Non-OP	1	0	None
	L	OP	1	0	None
4	R	OP	2	3	Yes
	L	Non-OP	2	3	Yes
5	R	OP	0	0	Yes
	L*	OP	0	0	Yes
6	R	OP	2	2	Yes
	L	OP	3	2	Yes
7	R	OP	0	0	None
	L	Non-OP	0	0	None
8	R	OP	0	1	None
	L	Non-OP	0	1	None
9	R	OP	1	0	None
	L	OP	0	0	None
10	R	OP	0	1	Yes
	L	Non-OP	0	1	Yes
11	R	Non-OP	1	1	None
	L	OP	0	1	None
12	R	OP	0	3	Yes
	L	Non-OP	2	3	Yes

*R* right, *L* left, *OP* operated, *non-OP* non-operated

*Not included in analysis due to follow-up < 1 year

### Radiographic outcomes

**Fig. 4:**
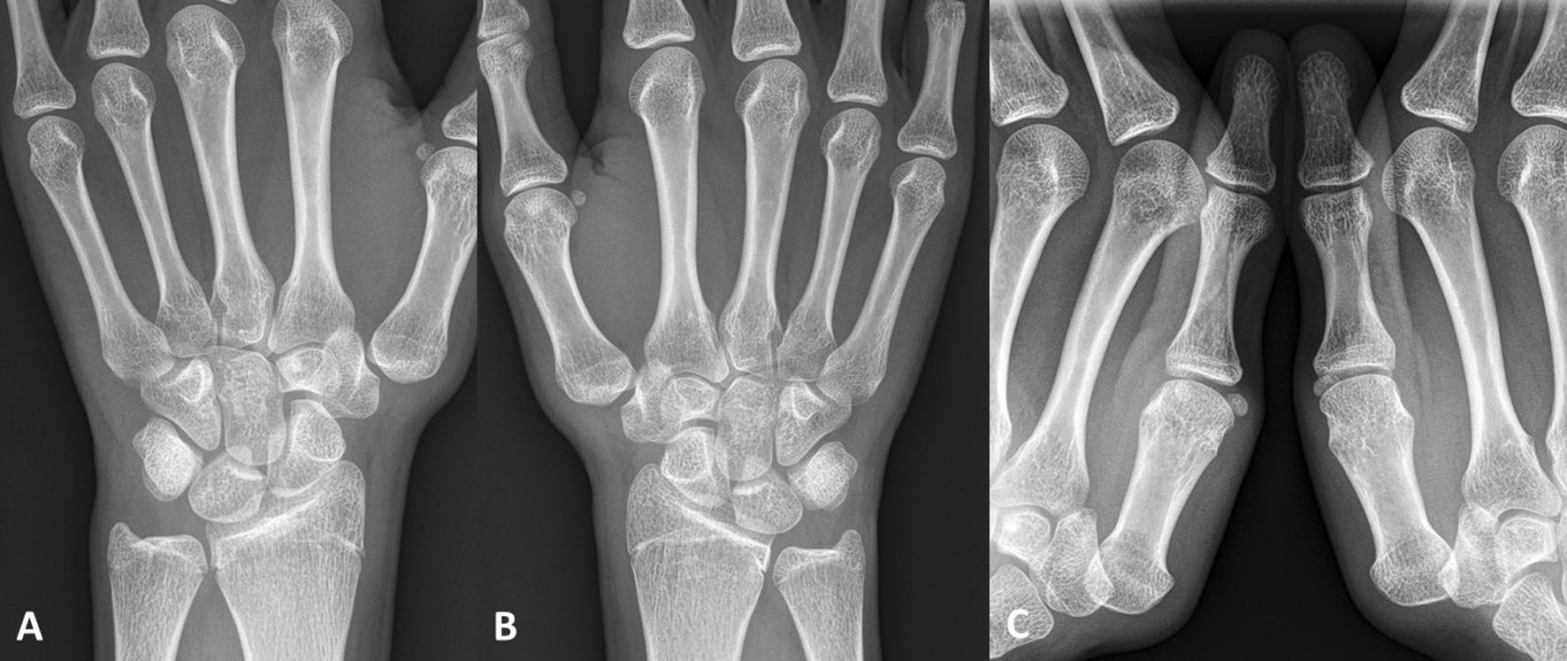
An adolescent patient with trapezium hypoplasia (**a, b**) and severe shifting (**c**) is shown. Nevertheless, she had no clinical symptoms at all on the operated right side

**Fig. 5:**
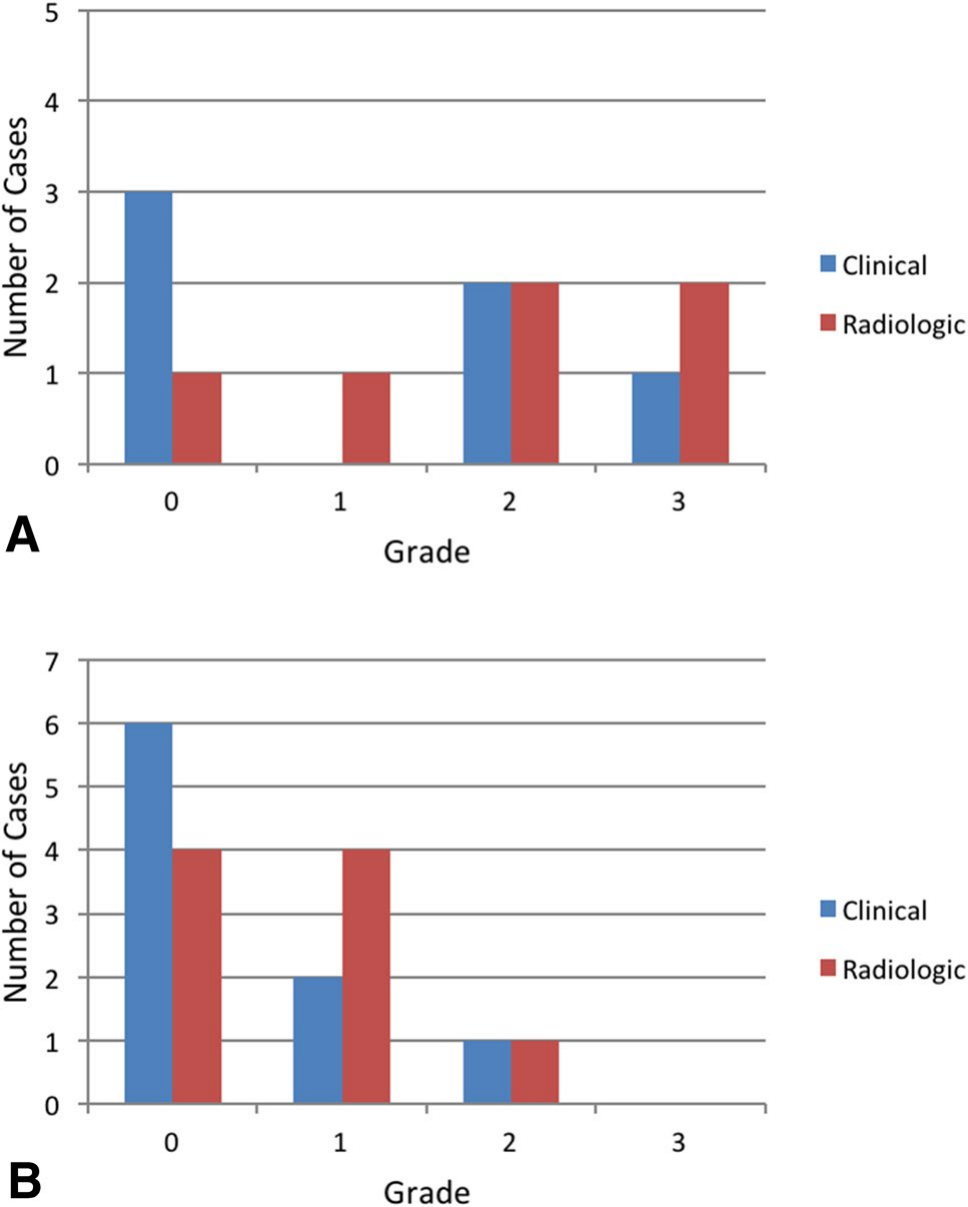
Stress tests are presented. **a** In patients with trapezium hypoplasia, **b** In patients without trapezium hypoplasia. Note the difference between groups with regard to grade 3 (severe) dislocations

Radiographic evaluation among the case cohort is shown in Table 2. In summary, five patients showed distinct signs of trapezium hypoplasia (Fig. 4) compared to seven patients with normal anatomy. Following surgery, four patients (five operated thumbs) presented without any radiographic signs of shifting compared to four cases (five thumbs) with mild, two (three thumbs) with moderate, and two (two thumbs) with severe signs during stress radiographs, respectively. As shown in Fig. 5, patients with trapezium hypoplasia tended to have higher grades of radiologic shifting (two of five cases [40%] with severe shift). As with clinical testing, there was no significant difference between operated and non-operated sides present after surgery (*p* = 0.776).

## Discussion

Hereditary joint hypermobility has been associated with the development of OA of the basal thumb joint, especially in women. This condition is often associated with pain, loss of grip and pinch strength, as well as functional limitations. Although the causal factor is not yet clear, the permitting of excessive ranges of movement due to band laxities and mechanical stresses on parts of cartilages not equipped for such a load due to subluxation are amongst the triggering factors for the development of OA of the CMC joint. With this regard, Jonsson et al. evaluated 100 patients (94 females and 6 males, mean age 66, range 41–78) with established hand OA who fulfilled the ACR criteria for hand OA and compared them with matched controls for the presence of joint hypermobility and OA of the thumb [5]. 39 patients compared to 32 in the control group displayed hypermobility features. This study group thus found that OA of the basal thumb in patients with hypermobility features was more common and more severe. Even patients with moderate laxity revealed more severe thumb base involvement and more disability. A statistical significance was shown for the correlation between the number of hypermobility criteria and disability [5]. However, OA not only occurs in the elderly, but may even be present in young patients. The joint hypermobility syndrome is estimated to occur in up to 36% of cases depending on diagnostic criteria. This genetic syndrome is characterized by an abnormal range of motion of various joints and is often associated with musculoskeletal pain after activities and intermittent joint swelling [12].

Therapeutic approaches to chronic pain of the basal thumb joint depend on preferences and expectations of the patients and vary due to comorbidities, which may negatively influence symptoms experienced by patients. Management of such symptoms often requires a combination of non-pharmacological, pharmacological and surgical approaches. Non-pharmacological approaches include splints, assistive devices, exercise and manual therapy. However, in patients with persistent chronic pain, surgical treatment should be considered [13].

Eaton and Littler first proposed the conventional technique of ligament reconstruction in CMC 1 instability in 1973 [6]. This procedure uses a slip of the FCR which is passed through the first metacarpal and wrapped around the tendons of the APL and FCR. Even though satisfactory stability of the joint is often achieved using this procedure, the CMC joint of the thumb has to be extensively exposed from its dorsal aspect to the wrist to accomplish the reconstruction. This approach increases the risk of iatrogenic injury to the dorsal sensory branches of the radial nerve during surgery. Iyengar et al. thereafter evaluated the efficacy of a modified Eaton–Littler procedure on clinical outcomes in patients with traumatic CMC 1 instability [14]. This technique uses a FCR tendon slip which is passed through an extra-articular bone tunnel of the metacarpal before being directed in an oblique manner to reproduce the anterior oblique ligament. The slip is then rerouted through the extra-articular bone tunnel and sutured back on itself. 11 patients who underwent this procedure were evaluated using QuickDASH scores, as well as pinch and grip strength measurements. Both showed statistical significant improvement after a mean follow-up of 6 years. Zhang et al. proposed an alternate technique using the radial half of the FCR tendon in traumatic CMC 1 instability [15]. The tendon is first weaved from radial to ulnar through a channel the trapezium and the metacarpal, and then wrapped around itself to be sutured to the insertion of the APL. The authors treated 13 patients using this procedure and observed no residual instability after a mean follow-up of 2 years. Friebel et al. lately studied the effectiveness of Arthrex Mini TightRope ligament reconstruction in an unstable trapeziometacarpal joint in anatomical models [16]. They included six fresh frozen arms from five cadavers to conduct their study after radiological examination for basal joint arthritis and joint instability. A significant improvement of ligament laxity was observed, leading to the conclusion that the Arthrex Mini TightRope provides a good stabilization without compromising range of motion. Finally, Langer et al. used a technique with an APL strip looped through a trapezium bone tunnel [3]. Among 24 patients, only one case of recurrent instability occurred. After 2 years of follow-up, no case of OA occurred among 11 patients. In contrast to the aforementioned techniques, the aim of our study was to evaluate the efficacy of a pure soft-tissue stabilization procedure using an APL tendon strip for CMC 1 stabilization. In our opinion, this technique bears less risk for complications such as impaired motion and no risk of trapezium fracture compared to bone tunnel techniques. Concerning stability, long-term results will show whether our soft-tissue technique can provide long-lasting stability as seen in the current mid-term follow-up.

With regard to incipient arthritic thumbs, several studies evaluated surgical stabilization techniques. Klein et al., for example, conducted a study to evaluate long-term results after modified Epping procedure in patients who suffered from trapeziometacarpal osteoarthritis [17]. After drilling a hole through the base of the first metacarpal, half of the flexor carpi radialis tendon is transected and passed through the hole. The FCR tendon piece is then tied around the APL tendon, led back into the trapezial void to create a sling around the APL tendon. Afterwards, the FCR tendon is sutured to the remaining FCR tendon and to the periosteum of the first metacarpal. Long-term results showed significant improvement of patient perceived pain during various activities, especially comparing pain during continuous motion and heavy manual work. Okita et al. reported a case in which they used suture anchor to reconstruct a traumatic dislocation of the carpometacarpal joint of the thumb [18]. Micro anchors were used to suture each ruptured ligament and the lateral capsule to its original position. The patient reported no pain, instability or functional disabilities at the 1-year follow-up. Radiological examination also showed a good position of the thumb with no post-traumatic changes.

In the current study, we included 12 patients who underwent surgery for chronic, habitual instability of the basal thumb joint without neither traumatic history nor OA. All patients had severe, painful clinical instability before surgery. Overall, patient satisfaction was high with excellent results in the reported DASH, Nelson and VAS scales. Only one case of recurrent instability was noted during the mid-term follow-up period. Nevertheless, a total of seven patients reported a VAS score between three and eight after repetitive, excessive strain on the CMC 1 joint. Moreover, stability was also restored when being assessed by stress test radiographs. During evaluation of the postoperative CMC 1 shift, which we divided into four groups (none, mild, moderate and severe), we noticed good clinical improvement of stability in the operated thumbs. A total of six operated thumbs still showed some residual clinical signs of mostly mild or moderate CMC 1 shift. Only two displayed a severe postoperative shift, one in the contralateral hand, which was not operated, and one, which had a recurrence of CMC 1 instability. Additionally, 10 of 15 operated thumbs showed some radiographic signs of shifting; however, these findings were mainly observed in cases with trapezium hypoplasia (Fig. 3), and were not significantly different compared to the healthy sides. Despite these radiographic findings, all patients except one were subjectively symptom free and satisfied. From the given findings, we may conclude that cases with trapezium hypoplasia may be prone for recurrent (radiologic) shifting, although this does not necessarily mean that these patients experience recurrent instability symptoms or pain.

This study reports the longest follow-up series evaluating clinical and radiographic results after a dedicated, easy-to-perform CMC 1 soft-tissue stabilization procedure using an APL tendon strip to date. Nevertheless, there are some shortcomings: first, the case cohort is somewhat limited in size. Second, 12 patients (50%) were not available for follow-up examination due to various reasons (mostly relocated), thus indicating a possible bias due to a loss to follow-up. Third, although women are known to be at the highest risk of hypermobility of the joint and subsequent dislocation and represent the vast majority (70–90%) of such cases, all but one patient in our case cohort were of female gender [5]. This, again, suggests that our results may have been biased due to a loss to follow-up of male patients. Hence, further research with a bigger sample size should be conducted.

In summary, our described technique was successful in restoring stability of the CMC 1 in the mid-term. Despite these promising results, future studies and follow-up should evaluate the durability in the long term, and specifically assess recurrence rates in patients with trapezium hypoplasia, as the lack of anatomic support to the CMC 1 may predispose for recurrent instability [19].
